# Prof. Boehm—Dissection Wound

**Published:** 1869-11

**Authors:** 


PROF. BOEHM—DISSECTION WOUND.—The *Union Medicale* of September 7, states that at that date Prof. Boehm, of Berlin, had probably ceased to live. Eight days before, he met with a slight dissection wound, of which he took no notice. Two days after, the hand became swollen, and fatal symptoms soon set in. The distinguished victim contemplated his fate with the utmost tranquility of mind.

